# Toxicity Evaluation of Graphene Oxide and Titania Loaded Nafion Membranes in Zebrafish

**DOI:** 10.3389/fphys.2017.01039

**Published:** 2018-01-04

**Authors:** Roberta Pecoraro, Daniele D'Angelo, Simona Filice, Silvia Scalese, Fabiano Capparucci, Fabio Marino, Carmelo Iaria, Giulia Guerriero, Daniele Tibullo, Elena M. Scalisi, Antonio Salvaggio, Isabella Nicotera, Maria V. Brundo

**Affiliations:** ^1^Department of Biological, Geological and Environmental Science, University of Catania, Catania, Italy; ^2^CNR-IMM, Catania, Italy; ^3^Department of Chemical, Biological, Pharmaceutical and Environmental Sciences, University of Messina, Messina, Italy; ^4^Department of Biology, University of Naples Federico II, Naples, Italy; ^5^Department of Biomedical and Biotecnological Sciences, University of Catania, Catania, Italy; ^6^Experimental Zooprophylactic Institute of Sicily, Catania, Italy; ^7^Dipartimento di Chimica e Tecnologie Chimiche, Università della Calabria, Rende, Italy

**Keywords:** *Danio rerio*, heme-oxygenase 1, inducible nitric oxide synthases, nano-composites, titanium dioxide

## Abstract

The use of nanomaterials in several application fields has received in the last decades a great attention due to their peculiar properties, but also raised many doubts about possible toxicity when these materials are used for some specific applications, such as water purification. Indeed a careful investigation is needed in order to exclude possible harmful side effects related to the use of nanotechnology. Nanoparticles effects on the marine organisms may depend on their chemical composition, size, surface structure, solubility, shape and how the individual nanoparticles aggregate together. In order to make the most of their potential, without polluting the environment, many researchers are trying to trap them into some kind of matrix that keeps them active but avoids their dispersion in the environment. In this study we have tested nanocomposite membranes prepared using Nafion polymer combined with various fillers, such as anatase-type TiO_2_ nanoparticles and graphene oxide. The non-toxicity of these nanocomposites, already shown to be effective for water purification applications in our previous studies, was recognized by testing the effect of the different materials on zebrafish embryos. Zebrafish was considered an excellent model for ecotoxicological studies and for this motivation zebrafish embryos were exposed to different concentrations of free nanoparticles and to the nanocomposite membranes. As biomarkers of exposure, we evaluated the expression of heme-oxygenase 1 and inducible Nitric Oxide Synthases by immunohistochemistry and gene expression. Embryo toxicity test showed that nor sublethal effects neither mortality were caused by the different nanoparticles and nano-systems tested. Only zebrafish larvae exposed to free nanoparticles have shown a different response to antibodies anti-heme-oxygenase 1 and anti- inducible Nitric Oxide Synthases. The immunolocalization analysis in fact has highlighted an increase in the synthesis of these biomarkers.

## Introduction

Nanotechnology has advanced exponentially over the past decade, and nanoscale materials being exploited in several applications (Tsuzuki, 2013). This growth of nanotechnology has not advanced without concerns regarding their potential adverse environmental impacts (Colvin, 2003, 2004; Dowling, 2004; Royal Society Royal Academy of Engineering., 2004; Warheit, 2004) and several nanotoxicology studies have been made in fact to e-value the toxicity of various nanoparticles (NPs) (Moore, 2006).

Engineered nanoparticles (NPs) represent an intermediate supramolecular state of matter between bulk and molecular material (Hoet et al., 2004). NPs biocompatibility surface properties depend on the charges carried by the particle and its chemical reactivity and size that giving a very large surface to volume ratio, The NPs size can be an extremely important factor for toxicity, and biodegradability (Brown et al., 2001; Hoet et al., 2004).

Because of the nanoscale nature of nanoscience and nanotechnology, they already bridge many fields including medicine, pharmaceuticals, manufacturing technologies, electronics and telecommunications (Perkel, 2003; Royal Society Royal Academy of Engineering., 2004; Kim et al., 2005).

The recent biomedical applications of graphene and derivatives have determined a rapid increase of the studies related to the biological interactions of these materials (Sanchez et al., 2012); for example graphene dispersed in air might represent a danger to people daily handling these materials, either by contact or inhalation, and studies on this topic are therefore necessary. Nevertheless, accidental spills and effluent discharges can determine an increased risk of release of these exogenous nanoparticles (NPs) into aquatic environment and even though emissions of graphene oxide (GO) to aquatic environment should be low (if any), their expected low degradability requires adequate investigation.

For graphene use are important to know the level of toxicity that it might reach in a biological system and the degree of safety; unfortunately, potential toxicity of graphene is little studied compared with that of other carbon nanostructures, such as carbon nanotubes (Seabra et al., 2014).

Nanoparticles in terrestrial organisms can be absorbed throughby inhalation or ingestion (Brigger et al., 2002; Moore, 2002; Colvin, 2003, 2004; Dowling, 2004; Warheit, 2004). Instead, in aquatic animals there are other routes of entry which gills and external surface epithelia (Moore, 2002). After absorption, nanoparticles are internalized occur via endocytosis (Na et al., 2003; Panyam and Labhasetwar, 2003; Panyam et al., 2003).

The toxic effects of Engineered Nanoparticles (ENPs) essentially depend on several key factors such as their intrinsic nature and capacity to form larger aggregations, the route of exposure, dose response, exposure time, the response of the receptor organisms to the lack of biocompatibility of ENPs, and the interactions in the mechanisms involved in the physiological process of uptake.

ENPs enter the environment via different exposure routes (Moore, 2002; Daughton, 2004; Moore et al., 2004; Royal Society Royal Academy of Engineering., 2004) and in natural water ecosystems ENPs can be degraded, transformed, carried and accumulated in a variety of ways. ENPs can form colloidal suspensions or can undergo processes of agglomeration or self-aggregation (Lapresta-Fernández et al., 2012), important factors that may influence their toxicity.

One of the most important factors related to the toxicity of ENPs is oxidative stress, with production of reactive oxygen species (ROS) (Lushchak, 2011).

In aquatic organisms, the effect of ROS on lipids can be measured by monitoring the intermediate species of lipid peroxidation and end products, that are tightly associated with exposure to NPs ROS-induced DNA damage may bring physiological consequences that impair reproduction (Guerriero et al., 2004, 2014), influences steroid-regulated physiology (Guerriero et al., 2005, 2017), inhibit growth, and damage both lysosomes and mitochondria. DNA damage leads to the liberation of toxic cations (Guo et al., 2008), induces ultrastructural alteration (Bartiromo et al., 2013), inhibits algal photosynthesis and affects electron and/or ion transport. The disruption of ion transport changes membrane permeability and increases the probability of NPs gaining entry inside the cell, which may lead ultimately to cell death.

The effects of ENPs on aquatic ecosystems are produced essentially by oxidative damage (internalization by cells) and interaction between ENPs and the cell membrane (without internalization) (Reyman et al., 2004). The properties of the NPs affect the uptake of NPs, which are bioaccumulated predominantly in the gills, intestine, liver (for larger NPs) (Scown et al., 2010), kidneys and blood. The latter three organs seem to be mainly due to internalization through the gills and the intestine. The main target of the toxicity mechanism is to disrupt the osmoregulatory function. Thus, the main adverse effects are related to an increase in the diffusive permeability of the membrane leading to a disturbed ion transport, as well as changes in cellular morphology, mitochondrial function, mitochondrial membrane potential and DNA damage-related gene expression that induce potential inflammation, necrosis and apoptosis of cells.

According to these results, ecotoxicology research is urgently required to explain and clarify the behavior of ENPs in their different forms and their environmental impact on the ecosystems. Fish Embryo Toxicity (FET) testis a modern non-animal test representing an effective alternative to acute test with adult fish (Embry et al., 2010; Pecoraro et al., 2017a). Fish embryo-larval assays, in fact, provide an investigative model that can be used for investigation of developmental toxicity mechanisms (Asharani et al., 2010; George et al., 2011; Ong et al., 2013; Brundo et al., 2016; Buccheri et al., 2016; Salvaggio et al., 2016; Xu et al., 2017).

Within the great variety of nanomaterials used for environmental applications, titania and graphene-based materials are extensively investigated (Scuderi et al., 2014). Graphene oxide (GO) and GO-based nanocomposites were recently proposed for the removal of pollutants (Yeh et al., 2013) and for adsorption of organics dyes from water (Sharma et al., 2013) and they are increasingly used for wastewater treatment in hybrid nanocomposite membranes (Filice et al., 2015). GO is highly dispersible in water and it shows semiconducting properties. The energy gap of GO can be tuned by a reduction of the oxygen functional groups making the valence and conduction band of GO suitable, respectively, for O_2_ and H_2_ evolution from water decomposition. The reduction of the oxygen content can be achieved by several ways, such as chemical or thermal treatments or UV light irradiation. Moreover, visible laser irradiation of GO flakes in water solution causes a modification of the oxygen content (D'Angelo et al., 2015) and size of the GO flakes. The conductive properties of reduced graphene oxide (RGO) become similar to that of pure graphene but with lower electron mobility (Mattevi et al., 2009). Graphene, GO and RGO showed also antibacterial properties (Liu et al., 2011; Buccheri et al., 2016).

Titania shows photocatalytic activity under UV light irradiation and TiO_2_ NPs are extensively used in the degradation of organic contaminant from water (Fujishima et al., 2000). Graphene Oxide (GO) flakes present an high capability to adsorb metal ions, can interact with microorganisms and therefore GO can be used in the wastewater treatments (Zhao et al., 2011; Buccheri et al., 2016). Recently, we have demonstrated (Filice et al., 2015) that Nafion-TiO_2_ under irradiation shows photocatalytic activity for degradation of Methyl Orange (MO) azo-dye with the formation of phenolic by-products. Nafion-GO_SULF_ is efficient like the Nafion-TiO_2_ membrane in the dye removal, without leaving the toxic by-products in the MO solution. Moreover, Nafion can be used as a polymer matrix in which TiO_2_, GO, and GO_SULF_ can be incorporated as nanoadditives, without any significant reduction of the photocatalytic efficiency with respect to the same fillers dispersed directly in solution (Filice et al., 2015).

In our paper we have evaluated the toxicity of Nafion based nanocomposites by zebrafish embryo toxicity test (ZFET), an alternative method to animal testing (Council Directive 86/609/EEC, 1986), that is considered a excellen test for the assessment of toxicity of nanocomposites (Brundo et al., 2016; Pecoraro et al., 2017a,b). As biomarkers of exposure, we analyzed Heme-Oxygenase 1 (HO1) and inducible Nitric Oxide Synthases (iNOS).

The materials tested in the present work are nanocomposite membranes prepared by dispersing various fillers, such as anatase-type TiO_2_ nanoparticles and graphene oxide (GO) in Nafion polymer. Furthermore, the ZFET results performed on the nanocomposite materials, were compared with results obtained for free TiO_2_ nanostructures and GO flakes dispersed as powder in the water solution.

## Materials and methods

### Materials

Nafion as a 20 wt% dispersion in water and lower aliphatic alcohols was supplied by Aldrich.

Graphene oxide in aqueous suspensions was synthesized by a modified Hummers' method (Hummers and Offeman, 1958), via oxidation processes of graphite powder using sulfuric acid, potassium permanganate and hydrogen peroxide.

Organo-modified GO (GO_SULF_) was prepared starting from GO produced by the Staudenmaier's method and then modified by using 3-amino-1- propanesulfonic acid, as described in a previous work (Enotiadis et al., 2012). Anatase TiO_2_ nanoparticles with a nominal average diameter of 21 nm, and methyl orange (MO, 0.1M in H_2_O) were acquired from Sigma-Aldrich.

### Membranes preparation

The preparation of hybrid nanocomposite Nafion membranes consists of the following steps: dispersing the fillers (anatase-type TiO_2_ nanoparticles and GO flakes) directly in Nafion solution, with a filler/polymer weight ratio of 3%, ultrasonicating for 1 day and stirring for another day at room temperature until a clear suspension is obtained. After that, the suspension was cast on a petri dish 50°C overnight to remove the solvents. Finally, the hybrid membrane is removed from the petri dish by immersing the glass plate in deionized water for several minutes. To reinforce the membrane, it is sandwiched and pressed between two Teflon plates and placed in oven at 150°C for about 25 min. All composite membranes produced by casting are subsequently treated by rinsing in: (1) boiling HNO_3_ solution (1 M) for 1 h to oxidize the organic impurities; (2) boiling H_2_O_2_ (3 vol%) for 1 h to remove all the organic impurities; (3) boiling deionized H_2_O for 40 min three times; (4) boiling H_2_SO_4_ (0.5 M) for 1 h to remove any metallic impurities; and again (5) boiling deionized H_2_O for 40 min twice to remove excess acid.

The membranes, as well as the fillers, were analyzed by scanning electron microscopy (SEM), using a ZEISS Supra 35 field emission SEM, in order to observe their morphology, homogeneity and size.

### Toxicity evaluation

#### Zebrafish maintenance and embryo collection

Zebrafish eggs fertilized within 4 h post fertilization (hpf) were provided from the Center of Experimental Ichthyiopathology of Sicily (CISS), University of Messina, Italy, and for experiments eggs were collected and chosen under a stereomicroscope (Leica M0205C, Multifocus). All embryos were derived from the same spawns of eggs.

### Fish embryo toxicity (FET) test

Fish Embryo Toxicity (FET) test was performed according to OECD (2013) and ISO 15088. Zebrafish embryos exposed to nanocomposite membranes (each one with a 1 cm^2^ area) and free TiO_2_ nanostructures and GO flakes at different concentrations (between 40 and 80 mg/L) in 5 ml of freshwater for 4–96 hpf were measured for toxic effects of a continuing observation period. The TiO_2_ and GO solutions were renewed and embryonic/larval mortality and hatching rate were evaluated every 24 h. As we described in previous paper (Brundo et al., 2016), healthy embryos were placed in 24-well culture plates (10 embryos in 5 ml solution/well). Each group had five replicate wells. Each experiment was replicated four times. During the exposure period, photographs of the embryos were made under a stereomicroscope (Leica M0205C, Multifocus) and the percentage of abnormal embryos was counted every 24 h.

### Immunohistochemical analysis

Some larvae were used for immunodetection of biomarkers by immunofluorescence. Non-specific binding sites for immunoglobulins were blocked by incubations for 1 h with normal goat serum (Vector Laboratories) in PBS (1:10) (Salvaggio et al., 2017).

The larvae were incubated overnight in a humid chamber at 4°C with the primary antibody anti-rabbit-heme-oxygenase 1 (1:500, Enzo Life Sciences, ADI-SPA-896) and anti-mouse-inducible Nitric Oxide Synthases (1: 500, Santa Cruz Biotechnology, Inc. Dallas, Texas USA, sc-7271). After a rinse in PBS for 10 min, the samples were incubated for 2 h at room temperature with fluorescein tetramethylrhodamine (TRITC) conjugated goat anti-rabbit IgG (1:1,000, Sigma-Aldrich) and fluorescein isothiocyanate (FITC) conjugated goat anti-mouse IgG (1:1,000, Sigma-Aldrich). Negative controls were performed by incubation with sera without antibodies. Observations were carried out using a microscope ZEISS AXIO Observer Z1 with Apotome2 system, equipped with the ZEN PRO software.

### Gene expression

Gene expression was performed by internal method, already described in Brundo et al. (2016). RNA was extracted by Trizol reagent (Invitrogen, Carlsbad, CA, USA). First strand cDNA was then synthesized with Applied Biosystem (Foster City, CA, USA) reverse transcription reagent. Quantitative real-time PCR was performed in 7900HT Fast Real-Time PCR System Applied Biosystems using the SYBR Green PCR MasterMix (Life Technologies). The primer sequences used are shown in Table 1. The specific PCR products were detected by the fluorescence of SYBR Green, the double stranded DNA binding dye. The relative mRNA expression level was calculated by the threshold cycle (Ct) value of each PCR product and normalized with that of β-actin 2 by using comparative 2−ΔΔCt method.

**Table 1:** Primer sequences used for gene expression assays.

**Gene**	**Primer forward**	**Primer reverse**
HO-1	ACGCTTACACCCGCTACCTC	ATCCCCTTGTTTCCAGTCAG
iNOS	CCTCCTCATGTACCTGAATCTCG	GCTCCTGCTTTAGTATGTCGC
β-actin2	AAGCAGGAGTACGATGAGTC	TGGAGTCCTCAGATGCATTG

### Statistical analysis

Statistical analysis was performed with Prism Software (Graphpad Software Inc., La Jolla, CA, USA). Data were expressed as ± SEM. Statistical analysis was carried out by unpaired *t*-test or ANOVA test to compare the means of more than two samples. The significance of differences between means was analyzed by ANOVA. *P* < 0.05 was considered statistically significant between experimental and control groups.

## Results and discussion

**Figure 1:**
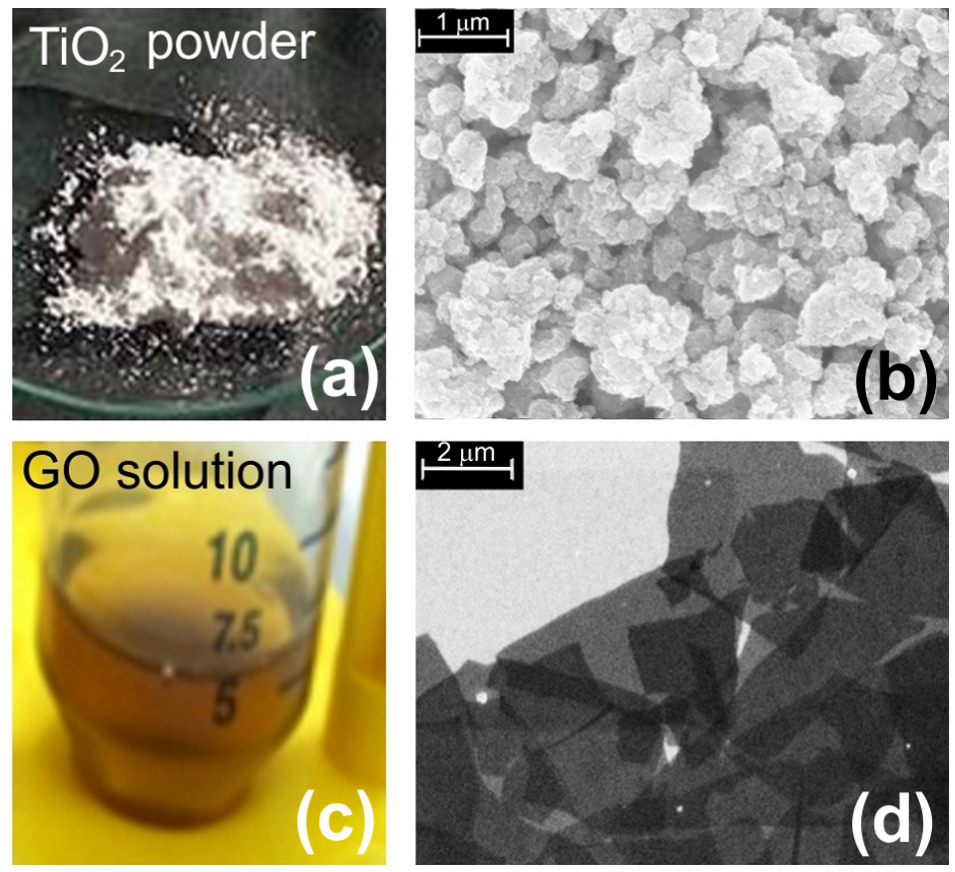
Image of nanomaterials. **(a)** Picture of TiO_2_ powder; **(b)** SEM image of aggregates of TiO_2_ nanoparticles; **(c)** a picture of GO dispersed in water solution; **(d)** SEM image of GO flakes.

In Figures 1a,c we report, respectively, the photos of the materials used for the present study both as fillers or as free particles: TiO_2_ nanoparticles and GO dispersed in water solution. SEM images of some aggregates of TiO_2_ nanoparticles and GO flakes deposited on silicon substrates are shown in Figures 1b,d.

**Figure 2:**
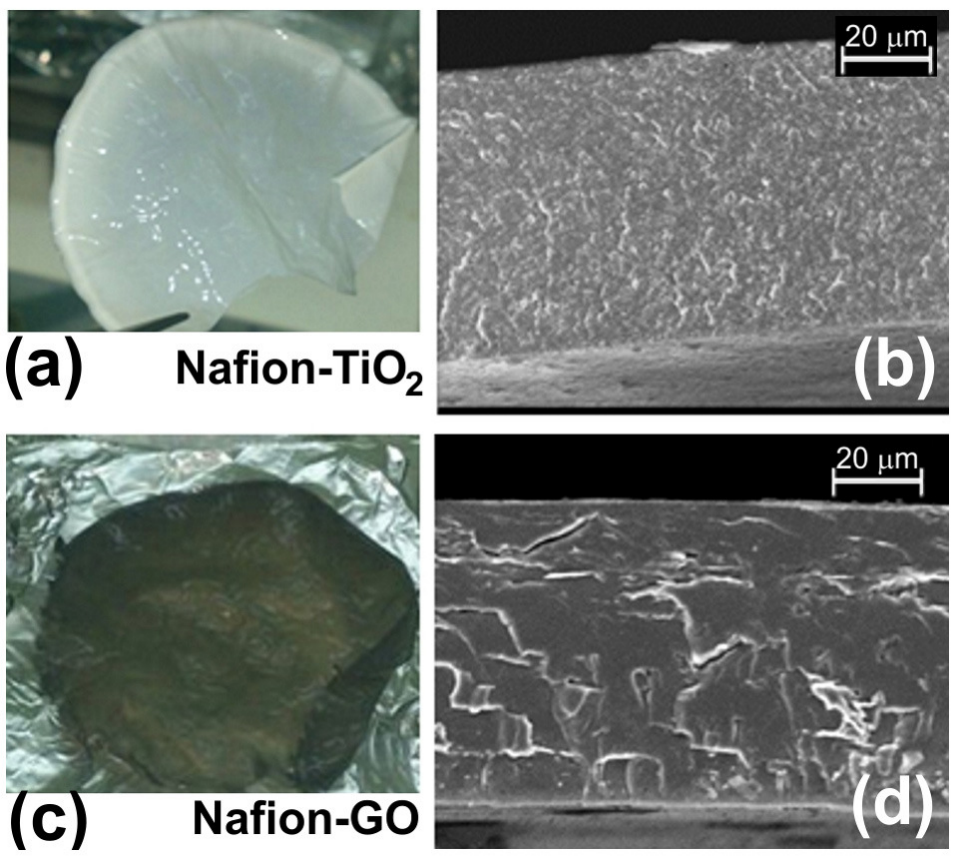
Image of Nanocomposite membranes. **(a)** a picture of a Nafion-TiO_2_ membrane and **(b)** SEM image of a cross section of the membrane; **(c)** a picture of a Nafion-GO membrane and **(d)** the same membrane observed by SEM in cross section.

Figures 2a,c show the nanocomposite membranes (Nafion-TiO_2_ and Nafion-GO, respectively) prepared by casting and Figures 2b,d report the same membranes observed by SEM in cross section. The fillers are homogeneously dispersed in the polymer, as also verified by energy dispersive X-ray analysis in a previous work (Filice et al., 2015).

**Figure 3:**
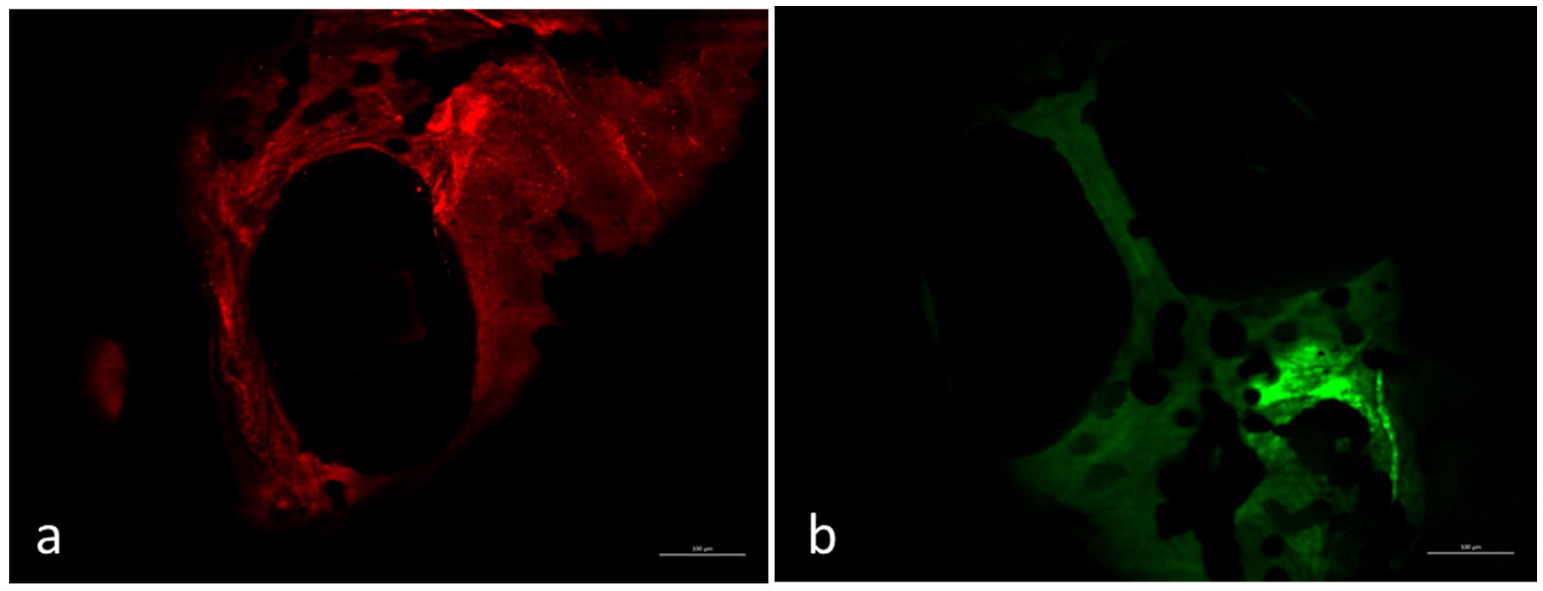
Larvae zebrafish 96 hpf free GO treated. Zebrafish exposed to free GO showed a positive response to anti-HO-1 **(a)** and anti-iNOS **(b)** in whole body. Scale bar: 150 μm.

**Figure 4:**
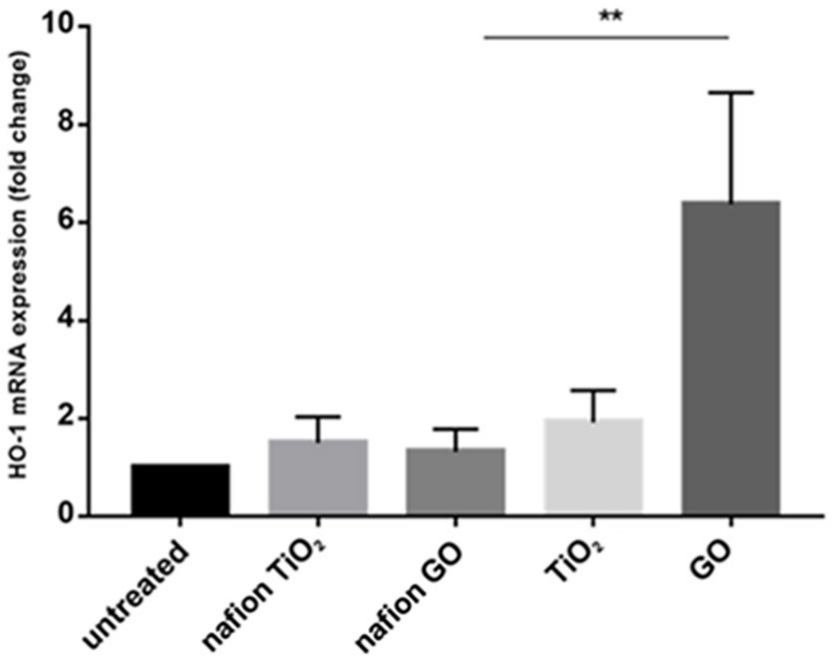
mRNA gene expression of HO-1 in zebrafish after exposure to free nanoparticles and nanocomposites. The HO-1 mRNA expression was increased only in free GO treatment. Bars represent the mean ± SEM of three independent experiments. ^**^P < 0.05 vs. untreated sample. (Calculated value of 2−ΔΔCt in untreated sample was 1).

**Figure 5:**
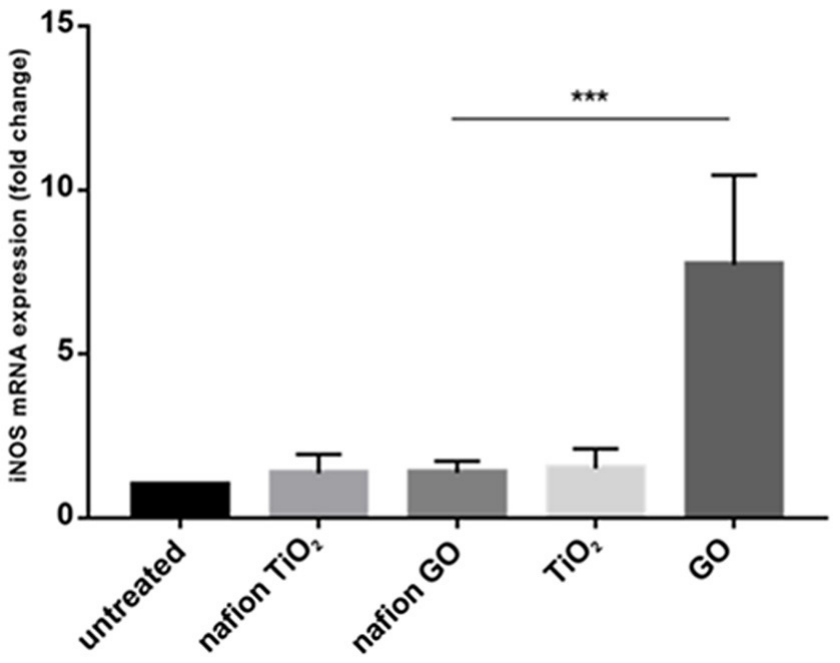
mRNA gene expression of iNOS in zebrafish after exposure to free nanoparticles and nanocomposites. The iNOS mRNA expression was increased only in free GO treatment. Bars represent the mean ± SEM of three independent experiments. ^***^P < 0.05 vs. untreated sample. (Calculated value of 2−ΔΔCt in untreated sample was 1).

Environmental safety of the nanocomposite materials and fillers was investigated through the ZTET, a modern toxicity test that representing an effective alternative to an acute test with adult fish. Zebrafish is considered an excellent animal model for the investigation of developmental toxicity mechanisms in environmental studies. ZFET revealed neither mortality nor sublethal effects caused by the different nanocomposites and free nanoparticles tested. In particular, no one of the evaluated endpoints (viability, growth, brain morphology, pharyngeal arches and jaw, heart, fins, notochord, somites, body shape, cardiovascular function, yolk sac and locomotor function) were satisfied. Significant differences were detected, conversely, in the expression of biomarkers in larvae exposed to the nanocomposites or to the free nanoparticles. Immunohistochemical analysis, in fact, performed in larvae exposed to nanocomposite membranes, did not show the presence of biomarkers, as well as control samples. Vice versa, the larvae exposed to GO flakes and TiO_2_ NPs showed a positive response to anti-HO1 and iNOS in the whole body (Figures 3a,b). These results were confirmed by gene expression analysis (Figures 4, 5).

## Conclusion

In this work, we evaluated the toxicity of nanocomposite membranes prepared using Nafion polymer combined with anatase-type TiO_2_ nanoparticles and graphene oxide. The analyses were also carried out with free TiO_2_ nanoparticles and GO flakes.

The results confirmed the non-toxicity of these nanocomposites, already shown to have great potential in eco-friendly water/wastewater purification in our previous studies (Filice et al., 2015; Buccheri et al., 2016; Scalese et al., 2016), was established by testing the effect of the different materials on *Danio rerio* larvae, with FET test that is a new non-animal test.

## Author contributions

MB, RP, and FM have carried out the planning of experiments, have elaborated the data and have drafted the manuscript; FC and CI take care fish facilities; RP, ES, and DT have carried out experiments of immunohistochemical and gene expression analysis; AS and GG have revised the manuscript; DD, SS, SF, and IN have realized NPs/nanocomposite membranes and have revised the manuscript.

### Conflict of interest statement

The authors declare that the research was conducted in the absence of any commercial or financial relationships that could be construed as a potential conflict of interest.
